# In-hospital outcomes of cardiac tamponade in patients with pulmonary hypertension: A contemporary analysis

**DOI:** 10.1371/journal.pone.0312245

**Published:** 2024-10-31

**Authors:** Moises Abraham Vasquez, Samuel De Jesus Vasquez, Natacha Vargas, Christian Guilliod, Antonio Luna Alvarez, Beatriz Rivera, George Leonor, Yiannis S. Chatzizisis

**Affiliations:** 1 Department of Internal Medicine, University of Miami Miller School of Medicine/Jackson Memorial Hospital, Miami, Florida, United States of America; 2 School of Medicine, Pontificia Universidad Catolica Madre y Maestra, Santiago, Dominican Republic; 3 Department of Medicine, Department of Cardiovascular Diseases, University of Miami, Miami, Florida, United States of America; RWJBH: RWJBarnabas Health, UNITED STATES OF AMERICA

## Abstract

**Background:**

Cardiac tamponade (CT) has an atypical presentation in patients with underlying pulmonary hypertension (PH). Evidence regarding the impact of PH on CT in-hospital outcomes is lacking.

**Methods:**

We used the National Inpatient Sample database to identify adult hospitalizations with a diagnosis of CT between 2016 and 2020, using relevant ICD-10 diagnostic codes. Baseline characteristics and in-hospital outcomes were compared in patients with and without a PH. Multivariate logistic regression analyses and case-control matching were performed, adjusting for age, race, gender, and statistically significant co-morbidities between cohorts.

**Results:**

A total of 110,285 inpatients with CT were included, of which 8,670 had PH. Patients with PH tended to be older (66 ± 15.7) and female (52.5%), had significantly higher rates of hypertension (74% vs 65%), CAD (36.9% vs. 29.6%), CKD (39% vs 23%), DM (32.1%, vs. 26.9%), chronic heart failure (19.0% vs 9.7%) and COPD (26% vs 18%)(P<0.001 for all). After multivariate logistic regression, PH was associated with higher all-cause mortality (aOR 1.29; 95% CI: 1.11–1.49), higher rates of cardiogenic shock (aOR: 1.19; 95% CI: 1.01–1.41), ventricular arrythmias (aOR: 1.63; 95% CI: 1.33–2.01), longer length of stay (11 days vs 15 days), and higher total hospitalization costs ($228,314 vs $327,429) in patients presenting with CT. Despite pericardiocentesis being associated with lower in-hospital mortality, patients with PH were less likely to undergo pericardiocentesis (aOR: 0.77; 95% CI: 0.69–0.86).

**Conclusion:**

PH was associated to increased in-hospital mortality and a higher rate of cardiovascular complications in an inpatient population with CT. Pericardiocentesis was associated with reduced mortality in patients with CT, regardless of whether they had PH. However, patients with PH underwent pericardiocentesis less frequently than those without PH.

## Introduction

Pericardial effusions are a mortality predictor and a marker of disease progression and poor prognosis in patients with pulmonary hypertension (PH) [1–5]. Most pericardial effusions in pulmonary hypertension are not hemodynamically significant and can be treated conservatively, but large effusions occasionally lead to cardiac tamponade (CT). Cardiac tamponade typically causes diastolic collapse of the right atrium and right ventricle [6]. This occurs because high intrapericardial pressures force the thinner and more compliant walls of the right heart chambers to bow inward. Altogether, the absence of any chamber collapse (RV or RA) on transthoracic echocardiogram has a 90% negative predictive value for tamponade [6, 7]. However, in the context of PH, atypical presentations of CT can occur, characterized by absence of classic tamponade clinical and echocardiographic signs due to elevated right heart pressures. Isolated left atrial or ventricular collapse in echocardiography is often the only sign of tamponade in this population [8–10]. As a result, PH masks the traditional CT presentation, making diagnosis challenging and potentially delaying life-saving treatment [11–13].

Prior case series have described the diagnostic and therapeutic challenge of tamponade in patients with PH [8–15]. The impact of this interaction on in-hospital outcomes remains poorly described. Furthermore, these studies have focused on Pulmonary Arterial Hypertension (PAH) patients in particular, with little to no data surrounding other World Health Organization (WHO) PH groups. Therefore, we used a large, representative database to describe the in-hospital outcomes and trends in complications and drainage strategies in hospitalized patients with PH and CT.

## Methods

The National Inpatient Sample (NIS), made publicly available by the Healthcare Cost and Utilization Project (HCUP) is the largest database of hospitalizations, and represents a 20%, random, stratified sample of hospital discharges in the United States. To identify the study population, International Classification of Diseases, 10th Revision, Clinical Modification (ICD-10-CM) diagnostic and procedural codes were used. Institutional review board approval was not needed, as all patient information is de-identified and publicly available. Guidelines according to the Strengthening the Reporting of Observational Studies in Epidemiology Statement were followed, along with methodologies for best practices with the use of claims datasets [16].

The NIS database was sampled from 2016 to 2020 to identify adult inpatients with a diagnosis of CT. The study sample was stratified into patients with and without concurrent PH. NIS discharge weights were used to achieve national estimates. For each hospitalization, we extracted the patients’ baseline demographic and clinical characteristics and hospital characteristics. Demographic variables included age, biologic gender, race/ethnicity (White, Black, Hispanic, other), along with data on the type of admission (elective/nonelective). Hospital characteristics included location/teaching status (rural, urban nonteaching, or urban teaching) and bed size (small, medium, or large). ICD-10-CM codes used to define comorbidities are provided in S1 Table.

The primary outcome of interest was in-hospital mortality. Secondary outcomes included in-hospital cardiogenic shock, cardiac arrest, ventricular arrhythmias, mechanical circulatory support use, vasopressor use, acute respiratory failure, and acute kidney injury (AKI). We also evaluated hospital length of stay (LOS) and total hospital costs. Frequency of tamponade drainage strategies (pericardiocentesis and pericardial window) were also compared between the two groups. When available, previously validated ICD-10-CM codes were used to define the baseline characteristics and in-hospital outcomes, consistent with those used in published reports [17–19].

Categorical variables were compared using the Pearson Chi Squared (χ2) test. Continuous variables were compared using independent samples Student t test if normally distributed, and Mann-Whitney U test for non-normally distributed continuous variables. Categorical values were recorded as number (percentage), and continuous variables were recorded as mean [standard deviation]. Weighted data were used for all statistical analyses. Missing demographic data were replaced using imputation to the dominant category. To account for differences in samples, adjusted odds ratios (aORs) for binary variables were obtained by multivariate analysis, adjusting for age, sex, and identified relevant comorbidities. Associations were expressed using aORs and 95% confidence interval (CI). The adjustment variables were selected based on their clinical significance that may directly influence the in-hospital outcomes. A secondary analysis was performed using a case-control matching method to match the hospitalizations with a diagnosis of CT and PH to those without PH to a 1:1 ratio. Variables used for matching and adjusting in the regression model are listed in S2 Table. To evaluate temporal changes in tamponade drainage strategies, annual frequencies of pericardiocentesis or pericardial window procedures among all hospitalizations with a diagnosis of cardiac tamponade were calculated. Cochran-Mantel-Haenszel trend test was used for categorical variables and linear regression was used for continuous variables. In accordance with the HCUP data use agreement, variables that contained a small number of observed (i.e., unweighted) hospitalizations (<11) were not reported to avoid the risk of person identification or data privacy violation [20]. Missing demographic data were replaced using imputation to the dominant category. Level of statistical significance was defined as p < 0.005. All statistical analyses were performed using SPSS (IBM SPSS Statistics, Version 28.0, IBM Corporation, Armonk, NY).

## Results

**Fig 1:**
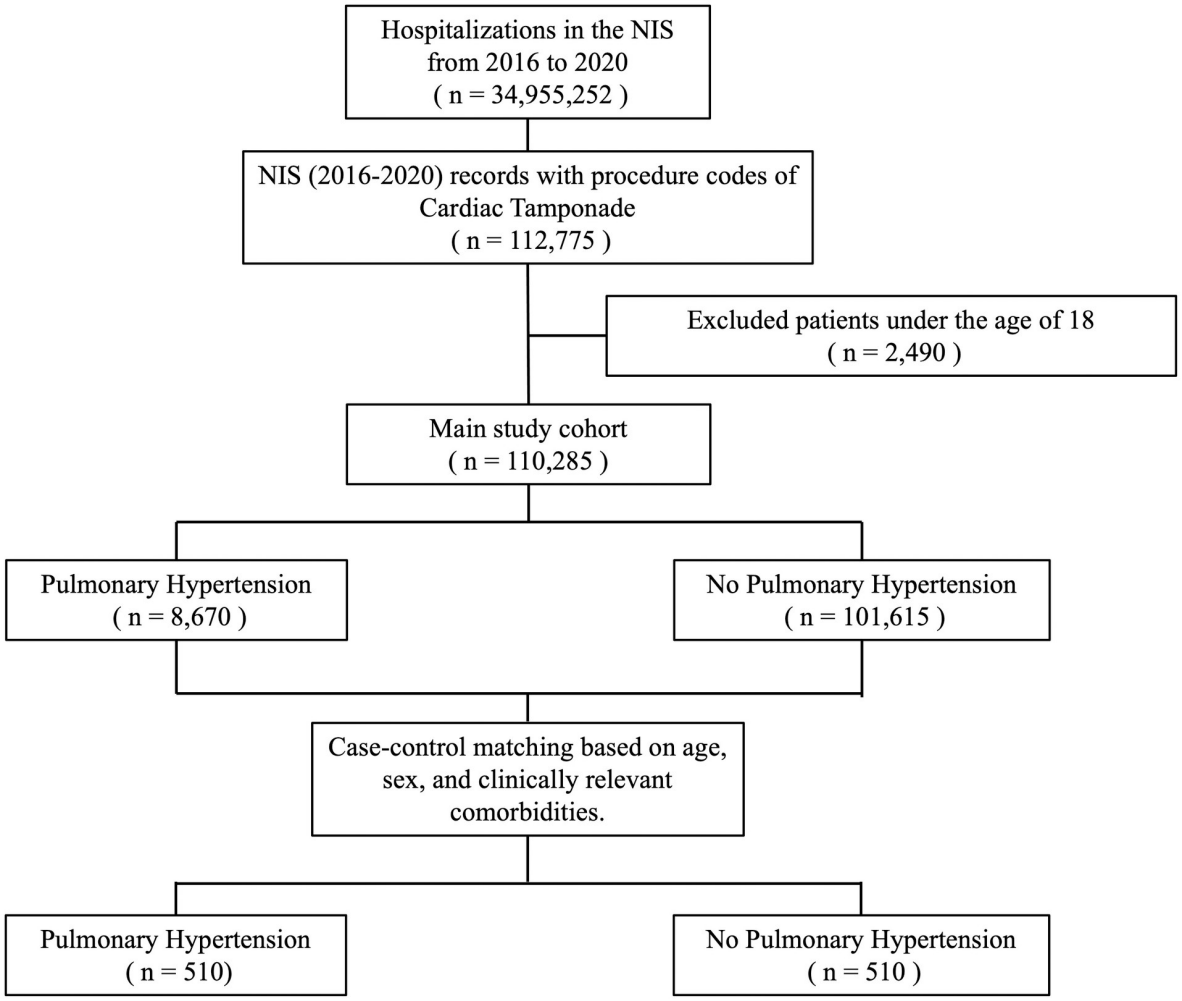
Study flow diagram showing inclusion and exclusion criteria. Hospitalization counts represent national-level estimates.

A total of 110,285 weighted hospitalizations with a diagnosis code of CT were identified between 2016 and 2020. Of these, 8,670 (7.7%) had a diagnosis of PH and 101,615 (93.3%) did not (Fig 1).

Patients with World Health Organization (WHO) PH Group 1 or Pulmonary Arterial Hypertension (PAH) represented 1.3% of the sample, with 110 observations. The total of patients with WHO PH Group 2, Group 3 and Group 4 were 290 (3.4%), 140 (1.6%), and 90 (1.0%) respectively. A total of 8,040 (93.3%) PH patients did not have a specified WHO PH category. Patients with tamponade and PH tended to be older (66 ± 15.7 vs. 63.1 ± 15.8 years; p <0.001) and female (52.5% vs. 46.2%; p <0.001) compared to patients without PH. The predominant race in both groups was White (63.6 vs 69.9%; p<0.001); the proportion of Black patients was significantly higher in the PH group (20.9% vs. 13.7%; p <0.001). Patients with CT and PH also had a higher baseline comorbidity burden such as chronic heart failure (19.0% vs. 9.7%; p < 0.001), chronic pulmonary disease (30.9% vs. 23.2%; p <0.001), chronic kidney disease (42.2% vs. 25.6%; p < 0.001), and coronary artery disease (37.6% vs. 29.3%; p <0.001). On the other hand, patients without PH had a greater proportion of patients with malignancy (75.0% vs 69.6%; p<0.001). The baseline characteristics of the cohort stratified by the presence of PH are shown in Table 1.

**Table 1:** Baseline characteristics in patients with cardiac tamponade, with and without pulmonary hypertension.

Variable	No PH, n = 101,615 (%)	PH, n = 8,670 (%)	P value
Age, years	63.1±15.8	66.0±15.7	<0.001
Female	46,965 (46.2)	4,550 (52.5)	<0.001
Race			
White	68,605 (69.9)	5,300 (63.6)	<0.001
Black	13,485 (13.7)	1,740 (20.9)	
Hispanic	9,425 (9.6)	700 (8.0)	
Other Races	6,575 (6.7)	590 (7.1)	
**Hospital characteristics**			
Relative bed size category of hospital			
Small	12,510 (12.3)	965 (11.1)	< 0.001
Medium	24,815 (24.4)	1,995 (23.0)	
Large	64,290 (63.3)	5,710 (65.9)	
**Location/teaching status of hospital**			
Rural	3,370 (3.3)	280 (3.2)	<0.001
Urban nonteaching	13,940 (13.7)	1,015 (11.7)	
Urban teaching	84,305 (83.0)	7,375 (85.1)	
Elective Admission	15,610 (17.8)	1,400 (18.1)	<0.001
**Comorbidities**			
Diabetes Mellitus	27,795 (27.6)	2,795 (32.50	<0.001
Obesity	18,705 (18.4)	1,815 (20.9)	<0.001
Hypertension	69,100 (68.0)	6,715 (77.5)	<0.001
Hyperlipidemia	41,385 (40.7)	3,770 (43.5)	<0.001
Smoking	38,295 (37.7)	2,630 (30.3)	<0.001
CAD	29,785 (29.3)	3,260 (37.6)	<0.001
PVD	11,860 (11.7)	955 (11.0)	0.067
Atrial Fibrillation	38,885 (38.3)	4,615 (53.2)	<0.001
Chronic Heart Failure	9,790 (9.7)	1,600 (19.0)	<0.001
CKD	25,695 (25.6)	3,630 (42.2)	<0.001
Dialysis Dependent	5,185 (5.1)	700 (8.1)	<0.001
Chronic Liver Disease	14,985 (14.7)	1,470 (17.0)	<0.001
Chronic Pulmonary Disease	23,590 (23.2)	2,680 (30.9)	<0.001
Hypothyroidism	14,700 (14.7)	1,430 (16.7)	<0.001
Anemia	29,540 (29.1)	3,155 (36.4)	<0.001
Coagulopathy	11,075 (11.0)	1,305 (15.2)	<0.001
Malignancy	26,520 (75.0)	1,065 (69.6)	<0.001
**Prior History**			
Prior MI	6,295 (6.2)	605 (7.0)	0.004
Prior PCI	6,915 (6.8)	535 (6.2)	0.024
Prior stroke	6,940 (6.8)	800 (9.2)	<0.001

CAD = Coronary artery disease, PVD = Peripheral Vascular Disease, CKD = Chronic Kidney Disease, MI = myocardial infarction, PCI

Baseline characteristics after case-control matching are shown in Table 2.

**Table 2:** Baseline characteristics in patients with cardiac tamponade, with and without pulmonary hypertension after case-control matching.

Variable	No PH, n = 510(%)	PH, n = 510 (%)	P value
Age, years	61.6±15.0	62.4±15.4	0.458
Female	315 (61.8)	315 (61.8)	1.000
Race			
White	400 (78.4)	400 (78.4)	1.000
Black	75 (14.7)	75 (14.7)	
Hispanic	25 (4.9)	25 (4.9)	
Other Races	10 (2.0)	10 (2.0)	
**Hospital characteristics**			
Relative bed size category of hospital			
Small	35 (12.3)	35 (12.3)	1.000
Medium	135 (24.4)	135 (24.4)	
Large	340 (63.3)	340 (63.3)	
**Location/teaching status of hospital**			
Rural	5 (1.0)	5 (1.0)	1.000
Urban nonteaching	85 (26.5)	85 (26.5)	
Urban teaching	420 (82.4)	420 (82.4)	
Elective Admission	40 (7.8)	40 (7.8)	1.000
**Comorbidities**			
Diabetes Mellitus	45 (8.8)	45 (8.8)	1.000
Obesity	45 (8.8)	45 (8.8)	1.000
Hypertension	255 (50.0)	255 (50.0)	1.000
Hyperlipidemia	130 (25.5)	130 (25.5)	1.000
Smoking	200 (39.2)	200 (39.2)	1.000
CAD	30 (5.9)	30 (5.9)	1.000
PVD	15 (2.9)	15 (2.9)	1.000
Atrial Fibrillation	200 (39.2)	200 (39.2)	1.000
Chronic Heart Failure	<10 (<2.0)	<10 (<2.0)	1.000
CKD	35 (6.9)	35 (6.9)	1.000
Dialysis Dependent	<10 (<2.0)	<10 (<2.0)	1.000
Chronic Liver Disease	15 (2.9)	15 (2.9)	1.000
Chronic Pulmonary Disease	150 (29.4)	150 (29.4)	1.000
Hypothyroidism	45 (8.8)	45 (8.8)	1.000
Anemia	130 (30.4)	130 (30.4)	1.000
Coagulopathy	10 (2.0)	10 (2.0)	1.000
Malignancy	380 (74.5)	380 (74.5)	1.000
**Prior History**			
Prior MI	<10 (<2.0)	<10 (<2.0)	1.000
Prior PCI	<10 (<2.0)	<10 (<2.0)	1.000
Prior stroke	10 (2.0)	10 (2.0)	1.000

CAD = Coronary artery disease, PVD = Peripheral Vascular Disease, CKD = Chronic Kidney Disease, MI = myocardial infarction, PCI = Percutaneous Coronary Intervention

The estimated overall in-hospital mortality rate was 14.5%, with statistically higher unadjusted rates in patients with PH compared to those without PH (17.3% vs 14.3%; p <0.001). Patients with PH had significantly higher rates of cardiovascular complications (34.8% vs 24.9%; p<0.001), including ventricular arrhythmias (73.8% vs 67.9%; p <0.001) and cardiogenic shock (58.3% vs 52.1%; p <0.001), higher rates of acute respiratory failure (43.7% vs 35.4%; p <0.001) and AKI (48.4% vs 37.4%; p <0.001). Patients with PH more often required mechanical circulatory support (12.1% vs 6.3%; p <0.001), and vasopressor use (10.2% vs 7.4%; p <0.001). Patients with PH also had a longer hospital LOS (15 vs 11 days; p <0.01) and higher total costs ($327,429 vs $228,314; p <0.01). Those with PH also underwent less pericardiocentesis (40.3% vs 45.4%; p <0.001) and pericardial window procedures (25.9% vs 27.3%; p = 0.004) than those without PH.

When adjusted for differences in baseline comorbidities listed in Table 1 using a multivariate logistic regression, CT with concurrent PH was associated with greater all-cause in-hospital mortality (aOR 1.29; 95% CI: 1.11–1.49). The adjusted odds of cardiogenic shock (aOR: 1.19; 95% CI [1.01–1.41]), ventricular arrhythmias (aOR: 1.63, 95% CI: 1.33–2.01), and acute respiratory failure (aOR 1.45; 95% CI: 1.28–1.63) were also significantly higher in patients with CT and PH. After adjusting, odds of vasopressor use were significantly lower in patients with PH (aOR 0.64; 95% CI 0.48–0.86). Patients with PH also had lower adjusted odds of undergoing pericardiocentesis compared to patients without PH (aOR: 0.77; 95%; CI: 0.69–0.86). There was no difference in pericardial window procedures (aOR 1.05; 95% CI: 0.93–1.18).

When comparing hospitalizations with CT and without PH that underwent pericardiocentesis compared to those that did not, drainage of effusion was associated with decreased adjusted odds of mortality (aOR 0.56; 95% CI: 0.52–0.60). Similarly, when comparing hospitalizations with CT and concurrent PH that underwent pericardiocentesis compared to those that did not, drainage of effusion was also associated with decreased adjusted odds of mortality (aOR 0.55; 95% CI: 0.40–0.76).

**Fig 2:**
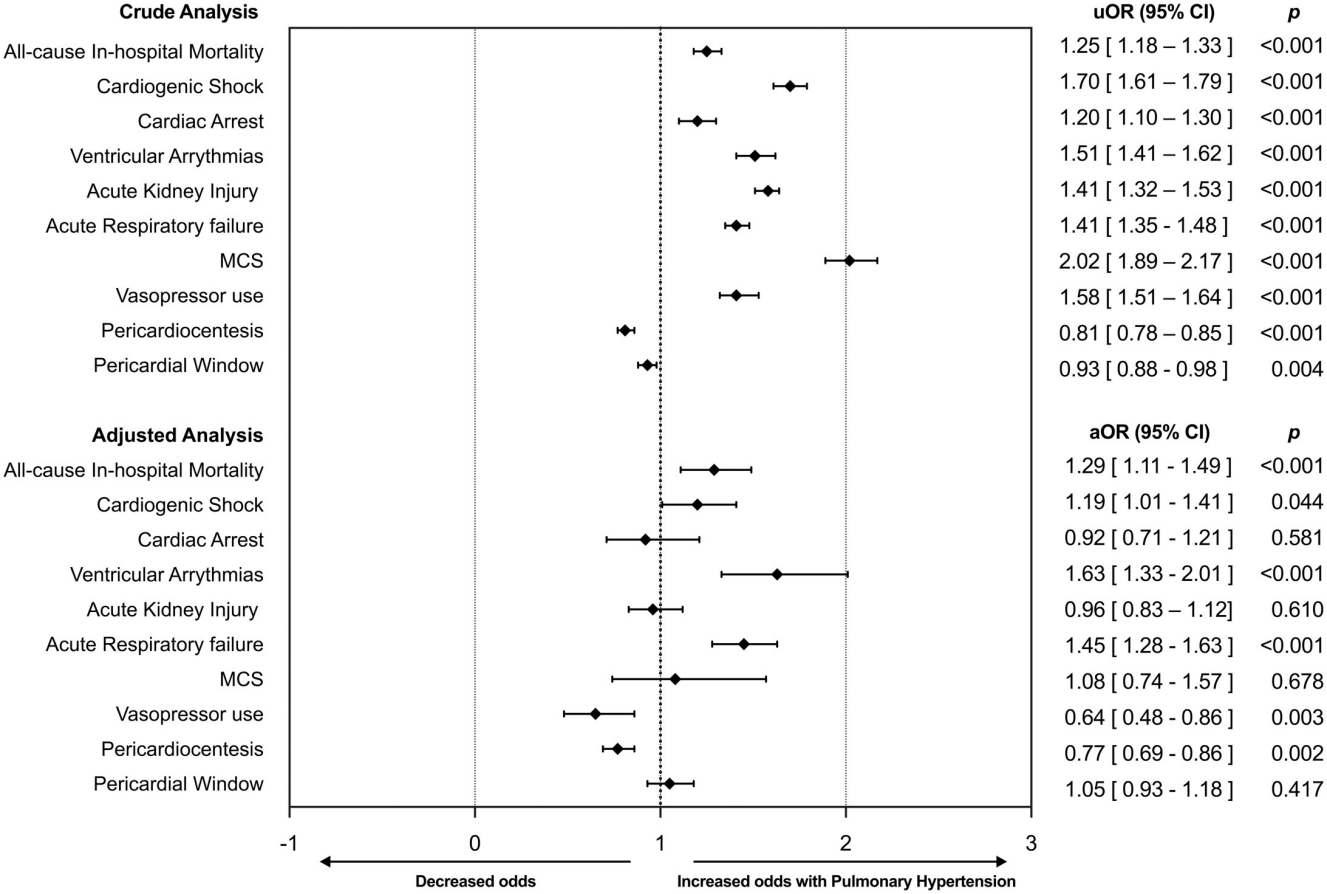
Forest plot showing crude and adjusted in-hospital outcomes in inpatients with cardiac tamponade and pulmonary hypertension compared to inpatients without PH. *Adjusted analysis based on age, gender, race, hospital location and teaching status, bed size, type of admission, and relevant co-morbidities. uOR = unadjusted odds ratio; MCS = Mechanical Circulatory Support.

After case-control matching with 1,020 matched patients (510 in each group), matched to all demographic characteristics and comorbidities listed in Table 1, PH was associated with higher odds of in-hospital mortality (OR 1.43; 95% CI: 1.02–2.01, p = 0.03), cardiac arrest (OR 2.43; 95% CI 1.31–4.51, p = 0.005), ventricular arrhythmias (OR 8.59; 95% CI 3.36–21.96, p < 0.001), acute respiratory failure (OR 2.07; 95% CI 1.60–2.66, p <0;001), and lower odds of undergoing pericardiocentesis (OR 0.76; 95% CI 0.59–0.97, p = 0.028) compared to patients without PH, confirming the findings of the multivariate regression analysis. The in-hospital outcomes stratified by the presence of PH are shown in Fig 2 and Table 3. The in-hospital outcomes after case-control matching are shown in Table 4.

**Table 3:** Main study outcomes of cardiac tamponade in patients with and without pulmonary hypertension.

	*No PH*, n = 101,615 (%)	*PH*, n = 8,670 (%)	p-value	Adjusted OR [95% CI]
**Primary Outcome**				
All-cause In-hospital Mortality	14,520 (14.3)	1,495 (17.3)	<0.001	1.29 [1.11–1.49]
**Secondary Outcomes**				
**Complications**				
Cardiogenic Shock	16,255 (16.0)	2,120 (24.5)	0.044	1.19 [1.01–1.41]
Cardiac Arrest	6,145 (6.0)	620 (7.2)	0.581	0.92 [0.71–1.21]
Ventricular Arrythmias	8,185 (8.1)	1,015 (11.7)	<0.001	1.63 [1.33–2.01]
AKI	37,960 (37.4)	4,200 (48.4)	0.610	0.96 [0.83–1.12]
Acute respiratory failure	35,945 (35.4)	3,790 (43.7)	<0.001	1.45 [1.28–1.63]
MCS use	6,445 (6.3)	1,045 (12.1)	0.678	1.08 [0.74–1.57]
Vasopressor use	7,540 (7.4)	885 (10.2)	0.003	0.64 [0.48–0.86]
**Drainage strategy**				
Pericardiocentesis	46,090 (45.4)	3,490 (40.3)	0.002	0.77 [0.69–0.86]
Pericardial Window	27,765 (27.3)	2,245 (25.9)	0.417	1.05 [0.93–1.18]
**Resource utilization**				
Mean LOS, days	11 [4–12]	15 [6–18]	<0.001	
Mean total charge, USD	$228,314 [$54,886 -$239,730]	$327,429 [$78,053 -$368,031]	<0.001	

*Adjusted for age, sex, race, hypertension, diabetes mellitus, chronic heart failure, coronary artery disease, hyperlipidemia, tobacco use, alcohol use, obesity, atrial fibrillation, dialysis dependent, chronic pulmonary disease, chronic liver disease, coagulopathy, malignancy, history of PCI, history of MI, history of stroke, hypothyroidism, anemia, hospital location and hospital teaching status

**Table 4:** Main study outcomes of cardiac tamponade in patients with and without pulmonary hypertension after case-control matching.

	*No PH*, n = 510 (%)	*PH*, n = 510 (%)	p-value	OR [95% CI]
**Primary Outcome**				
All-cause In-hospital Mortality	70 (13.7)	95 (18.6)	0.034	1.43 [1.02–2.01]
**Secondary Outcomes**				
**Complications**				
Cardiogenic Shock	30 (5.9)	40 (7.8)	0.216	1.36 [0.83–2.22]
Cardiac Arrest	15 (2.9)	35 (6.9)	0.005	2.43 [1.31–4.51]
Ventricular Arrythmias	<11 (<2.2)	40 (7.8)	<0.001	8.59 [3.36–21.96]
AKI	120 (23.5)	110 (21.6)	0.454	0.89 [0.66–1.19]
Acute respiratory failure	175 (34.3)	265 (52.0)	<0.001	2.07 [1.60–2.66]
MCS use	<11 (<2.2)	<11 (<2.2)	0.202	2.02 [0.68–5.95]
Vasopressor use	20 (3.9)	30 (5.9)	0.150	1.52 [0.85–2.73]
**Drainage strategy**				
Pericardiocentesis	265 (52.0)	230 (45.1)	0.028	0.76 [0.59–0.97]
Pericardial Window	175 (34.3)	175 (34.3)	1.000	1.00 [0.77–1.29]
**Resource utilization**				
Mean LOS, days	8 [4–8]	8 [5–12]	0.002	
Mean total charge, USD	$160,413 [$47,559 -$137,521]	$154,978 [$46,787 -$196,768]	0.828	

**Fig 3:**
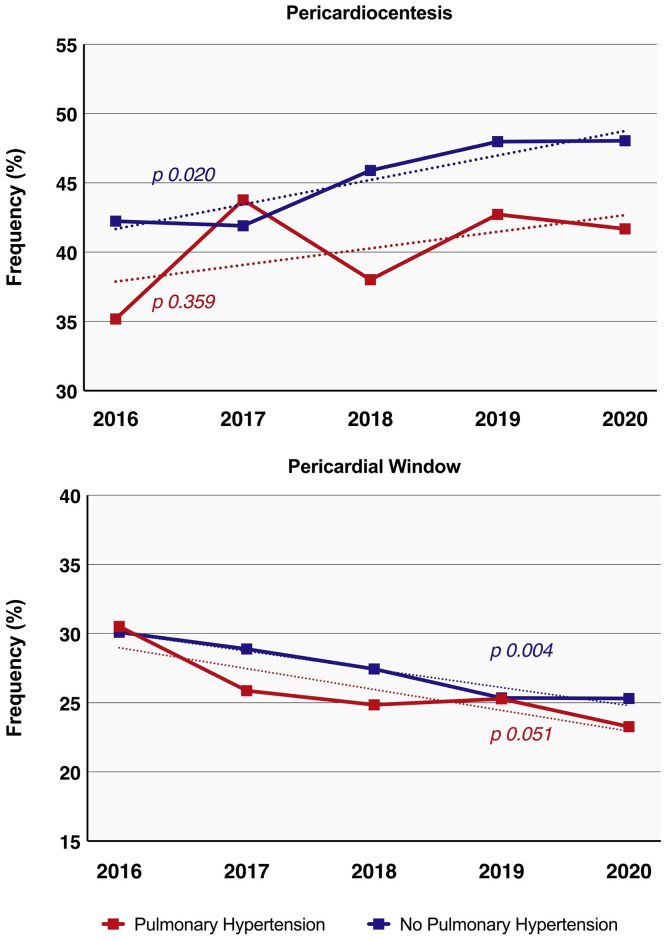
Temporal changes in frequency of pericardiocentesis (A) and pericardial window (B) procedures among adult inpatients with cardiac tamponade. Red line shows patients with tamponade and pulmonary hypertension. Blue line represents patients with tamponade and without pulmonary hypertension.

From 2016 to 2020, frequency of pericardiocentesis among all inpatients with CT significantly increased (41.6% in 2016 to 47.5% in 2020, p_trend_ = 0.015), however no significant trend was observed in patients with PH (35.2% to 41.7%, p_trend_ = 0.359) (Fig 3). There was a significant decrease in the rates of pericardial window procedures during the study period among inpatients without PH (30.1% in 2016 to 25.3% in 2020, p_trend_ = 0.004). There was no significant linear trend in pericardial window procedures in patients with PH (30.5% in 2016 to 23.3% in 2020, p_trend_ = 0.051). (Fig 3).

## Discussion

This study has several key findings: Firstly, among hospitalized patients with cardiac tamponade, the presence of PH was associated with higher rates of in-hospital mortality compared to those without PH. Secondly, the presence of PH was linked to a higher incidence of complications like cardiogenic shock, ventricular arrhythmias, and acute respiratory failure, as well as longer hospital stays and increased overall hospitalization costs compared to those without PH. Additionally, while pericardiocentesis was associated with lower mortality in CT in both groups, it was notably less frequently performed in patients with PH than in those without. Lastly, while there was a noticeable increase over time in pericardiocentesis procedures among patients with CT and without PH, this trend was not observed in patients with PH.

The negative prognostic significance of pericardial effusions in patients with PH, particularly in PAH, is well established [3–5]. Rarely, large effusions can progress to tamponade in PH, which are more often subacute [13]. In this context, right-sided chamber collapse is uncommon due to the increased pressure in the right heart chambers. Instead, the elevated right heart pressures push the interventricular septum into the left ventricle, causing the left-sided chambers to collapse, reducing preload, and further decreasing cardiac output [10–12, 21]. While this interaction is better understood in PAH, it is suggested that any form of PH that leads to right ventricular failure (Cor Pulmonale) could result in similar presentations of tamponade [6–8, 14, 15, 22–24].

The higher mortality in CT observed among patients with PH in our study is thought to be compounded by an increase in other adverse outcomes such as cardiogenic shock, ventricular arrythmias, and acute respiratory failure, in addition to the lower rate of pericardiocentesis performed in this group. These findings persisted after 1:1 case-control matching for cardiovascular and other relevant comorbidities. We hypothesize that due to the lack of florid signs of tamponade and the indolent nature of pericardial effusions in patients with PH, impending left ventricular failure is less frequently identified in these patients. This likely leads to progressive worsening of effusion, delayed recognition and treatment of tamponade in these patients, resulting in higher number of complications, and thus higher mortality [13–15]. Previous studies have also described how elevated right heart filling pressures and cardiac remodeling secondary to chronic pulmonary hypertension could trigger ventricular arrhythmias, consistent with our findings [25–27].

Given the subacute or chronic nature of pericardial effusions in the setting of PH, it has been proposed that these patients should be monitored periodically to prevent the development of tamponade [28–31]. Serial data from successive echocardiograms can serve as a basis for comparison and readiness for tamponade intervention if it eventually arises [28–31]. In such cases, pericardiocentesis, with close hemodynamic monitoring, should be performed without delay [32, 33]. A surgical approach (i.e. pericardial window) is preferred in certain situations; however, it does not improve clinical outcomes over pericardiocentesis and is associated with a higher rate of complications [32, 34].

Pericardiocentesis procedures were less frequently performed among inpatients with CT and PH compared to those without PH. This observation might be attributed to a deliberate choice in some cases to opt for conservative treatment, as pericardiocentesis has been associated with acute hemodynamic collapse and unacceptable periprocedural mortality in patients with PH and moderate to large pericardial effusions [18, 35–39]. In the setting of tamponade, however, intervention is warranted, but evidence of outcomes in this context is limited and inconsistent. Some studies have reported poor outcomes after pericardiocentesis among patients with PH and tamponade physiology, while others report no mortality or complications if appropriate precautions are taken during drainage [37–39]. In the absence of clear guidelines for drainage in patients with PH, it is possible that pericardiocentesis was avoided.

In a previous NIS study by our group that looked at in-hospital outcomes following pericardiocentesis in patients with and without PH, the presence of PH was associated with increased in-hospital mortality, post-procedure shock, and higher rates of cardiovascular complications in patients undergoing pericardiocentesis [18]. This previous study focused only on inpatients undergoing pericardiocentesis in the NIS and described the impact of PH on these outcomes. In contrast, the present study included inpatients in the NIS with a diagnosis of CT, stratified by the presence of PH. Our findings indicate that among inpatients with CT, pericardiocentesis is associated with reduced odds of mortality, regardless of the presence of PH. These results suggest that in the setting of CT, even with co-existing PH, undergoing pericardiocentesis is beneficial compared to a conservative approach. More frequent pericardiocentesis may potentially lead to reduced mortality rates and fewer complications in patients with CT and PH. Strategies to avoid hemodynamic deterioration during or after pericardiocentesis in these patients include gradual drainage of the effusion, swan-Ganz catheter monitoring of intracavitary pressures, and monitoring of intrapericardial pressure during the procedure [40]. Prospective studies are needed to further explore the impact of pericardiocentesis in patients with CT and PH.

### Limitations

This study has several limitations. Firstly, the data from the NIS database relies on ICD codes, which serve primarily for reimbursement claims and retrospective clinical summary. This method doesn’t provide detailed clinical presentation insights. Also, due to its retrospective nature, our study is susceptible to selection bias. The accuracy and consistency of coding in the NIS can vary, influenced by individual providers’ preferences and differences in documentation. Moreover, several potentially relevant data points are not captured in the NIS database. These include specifics like the severity of pulmonary hypertension, the size of pericardial effusion, detailed echocardiographic or other imaging data, hemodynamic parameters, medical therapy details, laboratory results, procedural specifics (such as criteria for deciding on drainage), and the timing of in-hospital events relative to admission. Additionally, there may be unmeasured or unknown confounding factors that could affect the outcomes. These factors include variations in management approaches, delays in pre-hospital care and hospital admission, and differences in treatment methods, all of which could lead to disparate outcomes among patient subgroups. Finally, the data reflect only a single hospital admission per patient, as the NIS does not include long-term follow-up information. Consequently, we were unable to consider out-of-hospital outcomes, including mortality and rehospitalizations. This aspect limits our ability to fully understand the long-term implications of the findings presented.

## Conclusion

We observed that PH was associated with increased in-hospital mortality and a higher incidence of cardiovascular complications among inpatients with cardiac tamponade. Furthermore, it was noted that pericardiocentesis was associated with reduced in-hospital mortality in cases of CT and PH. However, patients with PH underwent this procedure significantly less often compared to others. Prospective studies are needed to ascertain the benefit of pericardiocentesis procedures in patients with CT and PH.

## Supporting information

S1 Table ICD-10 diagnosis (CM) and procedure (PCS) codes used to identify comorbidities and outcomes. (DOCX)

S2 Table Variables used in multivariate regression analysis to compute adjusted odds of in-hospital outcomes and case-control matching model. (DOCX)

## Abbreviations

CT: Cardiac tamponade
PH: Pulmonary hypertension
