# Supplementary material for: In-hospital outcomes of cardiac tamponade in patients with pulmonary hypertension: A contemporary analysis
Source: PLoS One. 2024 Oct 31;19(10):e0312245. doi: 10.1371/journal.pone.0312245 (PMC11527273; doi:10.1371/journal.pone.0312245)
Supplement: S1 Table — (DOCX) [file pone.0312245.s001.docx]

**S1 Table.** ICD-10 diagnosis (CM) and procedure (PCS) codes used to identify comorbidities and outcomes.

| **Inclusion** | **ICD-10-CM/PCS codes** |
| --- | --- |
| Cardiac Tamponade | I314 |
| Pulmonary Hypertension | I27, I27.0, I27.1, I27.2x |

| **Baseline characteristics** | **ICD-10 CM codes** |
| --- | --- |
| **Comorbidities** | |
| Diabetes mellitus | E10.x, E11.x, E12.x E13.x |
| Hypertension | I10.x, I11.x-I13.x, I15.x |
| Nicotine/tobacco use | F17.x, Z72.0, Z87.891, Z7722 |
| Alcohol abuse | F10, Z72.1 |
| Obesity | E66.x, Z683x, Z684x, Z6854 |
| Coronary artery disease | I25.x |
| Peripheral vascular disease | I70.x, I71.x, I73.1, I73.8, I73.9, I77.1, I79.0, I79.2, K55.1, K55.8, K55.9, Z95.8, Z95.9 |
| A. fibrillation | I48.x |
| Heart failure | I50.x |
| Renal failure | N18.x, N19.x |
| Dialysis dependent | Z992, Z9115, Z4901, Z4902, Z4931, Z4932 |
| Liver disease | B18.x, B19.x, Z944, K9182, K70.x, K71.1, K71.3-K71.5, K71.7, K72.x-K74.x, K76.0, K76.2-K76.9. |
| Chronic pulmonary disease | I27.8, 127.9, J40.x-J47.x, J60.x-J67.x, J68.4, J70.1, J70.3 |
| Coagulopathy | D65-D68.x, D69.1, D69.3-D69.6 |
| Malignancy | C0x.x, C1x.x, C2x.x, C30.x, C31.x, C32.x, C33.x, C34.x, C37.x, C38.x, C39.x, C40.x, C41.x, C43.x, C45.x, C46.x, C47.x, C48.x, C49.x, C50, C51-58.x, C60-63.x, C76.x, C80.1, C81.x, C82.x, C83.x, C84.x, C85.x, C88.x, C9x.x |
| Anemia | D50x, D51x, D52x, D53x, D630, D631, D638, D649 |
| Hypothyroidism | E00.x-E03.x, E89.0 |
| **Previous history** | |
| Myocardial infarction | I25.2 |
| Stroke/TIA | Z86.73 |
| PCI | Z98.61, Z95.5 |

| **In-hospital outcomes** | **ICD-10 CM/PCS codes** |
| --- | --- |
| **Cardiovascular complications** | |
| Cardiogenic Shock | R570 |
| Post-procedural Shock | T8110XA, T8111XA,, T8119XA |
| Cardiac arrest | I46.x |
| Ventricular arrythmia | I4901, I4902, I472 |
| Vasopressor | 3E030XZ, 3E033XZ, 3E040XZ, 3E043XZ, 3E050XZ, 3E053XZ, 3E060XZ, 3E063XZ |
| **Other complications** | |
| Acute respiratory failure | J9620, J9621, J9622, J80, J9600, J9601, J9602, J9690, J9691, J9692 |
| Acute kidney injury | N17.x, N99.0 |
| Acute Liver injury | K7200, K7201 |
| **MCS** |  |
| Impella | 5A0211D, 5A0221D, 02HA3RS, 02HA3RZ, 02HA4RS, 02HA4RZ |
| BIVED | 02HA0QZ , 02HA0RJ, 02HA0RS, 02HA0RZ, 02HA3QZ, 02HA3RJ, , 02HA4QZ, 02HA4RJ |
| IABP | 5A02110, 5A02115, 5A02116, 5A02210, 5A02215, 5A02216 |
| ECMO | 5A0920Z, 5A15223, 5A1522F, 5A1522G, 5A1522H |
