# Supplementary material for: In-hospital outcomes of cardiac tamponade in patients with pulmonary hypertension: A contemporary analysis
Source: PLoS One. 2024 Oct 31;19(10):e0312245. doi: 10.1371/journal.pone.0312245 (PMC11527273; doi:10.1371/journal.pone.0312245)
Supplement: S2 Table — (DOCX) [file pone.0312245.s002.docx]

**S2 Table.** Variables used in multivariate regression analysis to compute adjusted odds of in-hospital outcomes and case-control matching model.

| **Demographic characteristics** |
| --- |
| Age |
| Biological sex |
| **Hospital characteristics** |
| Location/teaching status |
| Bed size |
| Elective admission |
| **Comorbidities** |
| Diabetes mellitus |
| Hypertension |
| Hyperlipidemia |
| Nicotine/tobacco use |
| Obesity |
| Coronary artery disease |
| Peripheral vascular disease |
| A. fibrillation |
| Chronic Heart failure |
| CKD |
| Dialysis dependent |
| Chronic Liver disease |
| Chronic pulmonary disease |
| Coagulopathy |
| Malignancy |
| Anemia |
| Hypothyroidism |
| **Previous history** |
| Myocardial infarction |
| Stroke/TIA |
| PCI |
